# Supplementary material for: Mitigating the impact of COVID-19 on primary healthcare interventions for the reduction of under-5 mortality in Bangladesh: Lessons learned through implementation research
Source: PLOS Glob Public Health. 2024 Mar 6;4(3):e0002997. doi: 10.1371/journal.pgph.0002997 (PMC10917255; doi:10.1371/journal.pgph.0002997)
Supplement: S1 Table — (DOCX) [file pgph.0002997.s004.docx]

#### S1 Table. Composition of key informants interviewed

| **Key informant representation** | **Department/Organization** |
| --- | --- |
| Ministry of Health & Family Welfare (MoH&FW)/Government: 8 (53.8%) | - Directorate General of Health Services - Directorate General of Family Planning - Bangabandhu Sheikh Mujib Medical University Hospital - Mohammadpur Fertility Services and Training Centre - Institute of Epidemiology, Disease Control and Research |
| Implementing partner/donor: 4 (30.7%) | - UNICEF - BRAC Bangladesh - Save the Children - United Nations Development Programme |
| Private sector: 1 (7.7%) | - Obstetrical and Gynecological Society of Bangladesh |
