# Supplementary material for: Mitigating the impact of COVID-19 on primary healthcare interventions for the reduction of under-5 mortality in Bangladesh: Lessons learned through implementation research
Source: PLOS Glob Public Health. 2024 Mar 6;4(3):e0002997. doi: 10.1371/journal.pgph.0002997 (PMC10917255; doi:10.1371/journal.pgph.0002997)
Supplement: S2 Table — (DOCX) [file pgph.0002997.s005.docx]

#### S2 Table. Transferable lessons

| **Transferable Lessons** | **Frequency of response** |
| --- | --- |
| Build in collaboration and strong coordination of activities | 6 |
| Invest in health systems, inputs, and quality | 5 |
| Engage with community and civil society | 5 |
| Use data for decision-making at all levels | 4 |
| Ensure effective leadership and commitment at all levels | 4 |
| Ensure public trust and building ownership in the health system | 2 |
| Develop strong guidelines and procedures, and timely communication | 1 |
| Leverage COVID-19 response to accelerate innovation | 1 |
| Strengthen data system (both public and private) | 1 |
