# Supplementary material for: Mitigating the impact of COVID-19 on primary healthcare interventions for the reduction of under-5 mortality in Bangladesh: Lessons learned through implementation research
Source: PLOS Glob Public Health. 2024 Mar 6;4(3):e0002997. doi: 10.1371/journal.pgph.0002997 (PMC10917255; doi:10.1371/journal.pgph.0002997)
Supplement: Attachment — Submitted filename: Point-by-point response - editorial assessment.docx [file pgph.0002997.s006.docx]

16 August 2023

Dear Editors,

Thank you for your initial editorial check and the opportunity to resubmit. We have addressed the edits required as point by point below:

1. Where possible, we encourage authors to provide relevant data underlying their research with their submission in order to facilitate editorial and peer review, even if the data may be restricted from publication or made private by a data repository prior to article acceptance. Many repositories permit private access for review purposes and have policies for public release at publication. For data concerning human research subjects, please anonymize the data as appropriate and follow pertinent legal and ethical protocols prior to sharing with editors and reviewers. If you are unable to share any pertinent data at the time of editorial and peer review, please kindly let the journal office know.

**Response:** The District Health Information System 2 (DHIS2) data is owned by the Bangladesh Directorate General of Health Services (DGHS) under the Ministry of Health and Family Welfare (MoH&FW). The Ethics approval letter specifically indicate: *“Data/samples should not be disclosed, made available to or use for purposes other than those specified in the protocol, and shall be preserved for a period, as specified under Centre's policies/practices (Ethical Review Committee (PR#21020, Date: 14 April 2021, document uploaded in this submission).”* We have therefore been unable to share the dataset publicly. We have now provided a statement under the data sharing section as below:

*“Data access is restricted to users with appropriate ethics approval from the committees listed in the Ethical Considerations section. Interested readers may apply to the authors for access by providing a written description of intended use. If the methodology does not violate the condition of informed consent under which the interview was conducted, and the proposal approved by UGHE, the user can obtain the data from the corresponding author.”*

2. Please complete all items on the Human Participants Research Checklist that are relevant for your submission, by following this link: <http://journals.plos.org/plosone/s/file?id=dc11/PLOS%20Human%20Participants%20Research%20Checklist.docx>.

**Response:** We have included the Human Participants Research Checklist and uploaded the ethics approval letter from the relevant body in Bangladesh.

Sincerely,

Dr. Alemayehu Amberbir M.P.H, Ph.D
