# Supplementary material for: Mitigating the impact of COVID-19 on primary healthcare interventions for the reduction of under-5 mortality in Bangladesh: Lessons learned through implementation research
Source: PLOS Glob Public Health. 2024 Mar 6;4(3):e0002997. doi: 10.1371/journal.pgph.0002997 (PMC10917255; doi:10.1371/journal.pgph.0002997)
Supplement: Attachment — Submitted filename: Response to editors.docx [file pgph.0002997.s007.docx]

7 December 2023

Dear Editors,

It is our pleasure to submit to you the revised article titled *Mitigating the impact of COVID-19 on primary healthcare interventions for the reduction of under-5 mortality in Bangladesh: lessons learned through implementation research.* Below we respond to the journal revision requests.

1. Please amend your detailed Financial Disclosure statement. This is published with the article. It must therefore be completed in full sentences and contain the exact wording you wish to be published.

a. State the initials, alongside each funding source, of each author to receive each grant.
b. State what role the funders took in the study. If the funders had no role in your study, please state: “The funders had no role in study design, data collection and analysis, decision to publish, or preparation of the manuscript.”
c. If any authors received a salary from any of your funders, please state which authors and which funders.

If you did not receive any funding for this study, please simply state: “The authors received no specific funding for this work.”

**We have revised the funding statement to read as follows: “This work was supported by Gates Ventures (AA, AB, LRH). The funders had no role in study design, data collection and analysis, decision to publish, or preparation of the manuscript.”**

2. Please provide separate figure files in .tif or .eps format only and remove any figures embedded in your manuscript file. Please also ensure all files are under our size limit of 10MB.
For more information about figure files please see our guidelines:
<https://journals.plos.org/globalpublichealth/s/figures>
<https://journals.plos.org/globalpublichealth/s/figures#loc-file-requirement>
**We have revised our three article figures and one supplemental figure accordingly and uploaded them in .tif format.**

3. In line with our goal of ensuring long-term data availability to all interested researchers, PLOS’ Data Policy states that authors cannot be the sole named individuals responsible for ensuring data access (<http://journals.plos.org/globalpublichealth/s/data-availability#loc-acceptable-data-sharing-methods>).

a) If there are ethical or legal restrictions on sharing a de-identified data set, please explain them in detail (e.g., data contain potentially identifying or sensitive patient information, data are owned by a third-party organization, etc.) and who has imposed them (e.g., a Research Ethics Committee or Institutional Review Board, etc.). Please also provide non-author contact information for a data access committee, ethics committee, or other institutional body to which data requests may be sent.

**We have added non-author contact information for the institutional body to which data requests may be sent. Our availability of data and materials statement now reads: “The data that support the findings of this study are available from the Directorate General of Health Services under the Ministry of Health and Family Welfare in Bangladesh (requests may be sent to** [**dg@ld.dghs.gov.bd**](mailto:dg@ld.dghs.gov.bd) **and** [**adgadmin@ld.dghs.gov.bd**](mailto:adgadmin@ld.dghs.gov.bd)**). However, restrictions apply to the availability of these data, which were used for the current study, and so are not publicly available. The datasets used and/or analyzed during the current study are available from the corresponding author on reasonable request and with permission of the Directorate General of Health Services under the Ministry of Health and Family Welfare in Bangladesh.”**

We thank you very much for your time and for your consideration of this research for publication in PLOS Global Public Health.

Sincerely,

Dr. Alemayehu Amberbir MPH, PhD

University of Global Health Equity

Kigali, Rwanda
