# Supplementary material for: Mitigating the impact of COVID-19 on primary healthcare interventions for the reduction of under-5 mortality in Bangladesh: Lessons learned through implementation research
Source: PLOS Glob Public Health. 2024 Mar 6;4(3):e0002997. doi: 10.1371/journal.pgph.0002997 (PMC10917255; doi:10.1371/journal.pgph.0002997)
Supplement: Attachment — Submitted filename: Response to reviewers _9 Feb 2024.docx [file pgph.0002997.s008.docx]

*Response to reviewers for PGPH-D-23-01582: Mitigating the impact of COVID-19 on primary healthcare interventions for the reduction of under-5 mortality in Bangladesh: lessons learned through implementation research*

| **Comment** | **Response** |
| --- | --- |
| Reviewer 3 | |
| 1. The title of the manuscript talks about the impact of COVID-19 on primary healthcare interventions. But it is not discussed in the text. It would also be good to describe Bangladesh’s primary healthcare delivery system | Thank you for taking time to review this article and for your feedback.  We agree that it is important to include context for Bangladesh’s primary healthcare delivery system. We have added the following to the background, page 4 lines 104-109: “The Ministry of Health and Family Welfare leads Bangladesh’s multifaceted health system and oversees two directorates: The Directorate General of Health Services (DGHS), primarily charged with managing public health services and providing general healthcare; and the Directorate General of Family Planning (DGFP), initially responsible for managing and providing family planning and components of maternal healthcare. Primary healthcare interventions including those contributing to essential health services for children under 5 take place under both directorates.”  We have further added “primary healthcare” to lines 34, 135, 153, 165, 179, 223, 267, 294, 389, and 414 in front of EBI (now, “primary healthcare EBIs”) in order to more explicitly describe the evidence-based interventions (EBIs) we considered in this work as the primary healthcare interventions that they are. |
| 2. Background: I would combine the first and fourth paragraphs (lines 101-105) and present them as a problem statement. I would also move the last sentence of the third paragraph (lines 99-100) to the first paragraph. | Thank you for your suggestion. We have made the changes that you have recommended, as shown in the track changes version of the document. |
| 3. Evidence showed that care-seeking for sick children rebounds later than other maternal and child health services. I was expecting care-seeking for sick children (such as pneumonia and diarrhea) as one of the key EBI for under-5 children to be explored in this study. | Thank you for your comment. We agree that care-seeking for sick children, including for pneumonia and diarrhea, are important EBIs for children under 5. We did include outpatient attendance for children, and found that there was a marked drop (about 56%) in outpatient department attendance for children under 5 in public health facilities nationally (IRR=0.443; 95%CI: 0.442, 0.444) between March to December 2020 as compared to the period of 2019, which is shown in Table 1 on page 10 (we added this table in response to previous reviewer feedback, so it may not have been in the version that you have reviewed; we have also included the table below for ease of reference). |
| 4. I am not sure the Poisson regression model researchers did is a true time series analysis method that would show pre-COVID, immediate, and post-COVID trends. I would report the monthly rate of decline in EBIs from the interrupted time series analysis (ITSA) output: 1) the pre-COVID-19 trend, 2) the immediate effect of COVID-19, and 3) the post-COVID-19 change (that is the differences between pre-COVID-19 and post-COVID-19 periods) on the EBI child health intervention coverage attributable to COVID-19 with counterfactual estimates of what would have happened without COVID-19, given the pre-existing trend of EBIs. For this purpose, researchers can use the “itsa” Stata command. Also, present model assumptions. | Thank you for your feedback. We opted to apply a slightly different approach using scatter plots using interrupted time series analysis. We used interrupted time series segmented regression analyses by fitting a Poisson regression model to estimate the impact of COVID-19 on childhood immunization, maternal health services (antenatal care visits and facility-based delivery) and outpatient attendance for children.  The model included a time variable, a dummy COVID-19 variable indicating pre-COVID-19 and the period of COVID-19, and primary health services reporting calendar months. We adjusted the model for seasonality using two pairs of sine and cosine terms (Fourier terms) included in the model. These are pairs of sine and cosine functions of time with an underlying period reflecting the full seasonal cycle (i.e. calendar year). We analyzed the data using Stata 16.1 (Stata-corp, College Station Texas, USA).  We have now elaborated our quantitative data analysis section to include the above text along with details of model specifications (Methods section, page 8, lines 203 to 211). We have also included our analysis findings from the Poisson regression model in the new Table 1 in addition to the scatter plot figures. The new Table 1 is at the bottom of this document for ease of reference. |
| 5. Figures 1 and 2 showed a decline trend before COVID-19 was first confirmed in the country. Can you comment what does it mean? | Thank you for your question. There was a slight decline just before the official report of COVID-19 in the country which could be due to the initial disinformation and media campaign related to COVID-19 globally even prior to the first cases of COVID-19 confirmed on March 8, 2020; however, issues of data quality including missing data and reporting errors in the DHIS2 dataset are difficult to exclude. Some amount of seasonal variability could also explain some of the trend.  We have added the following sentence to the discussion (page 23, lines 433-39) (please note that in a previous response to reviewers we added a new figure 1, so the figures in question are now #s 2 and 3): “Figures 2 and 3 showed fluctuating trends immediately before the COVID-19 period, with lower vaccines doses administered and lower facility deliveries in the month or months before the lockdown began. These slight declines just before the official report of COVID-19 in the country could be due to the initial disinformation and media campaign related to COVID-19 globally even prior to the first cases of COVID-19 confirmed on March 8, 2020; however, issues of data quality including missing data and reporting errors in the DHIS2 dataset are difficult to exclude. Some amount of seasonal variability likely also explains some of the trend.” |
| 6. Minor comments; Line 78, add “,” after the word pandemic; line 239, spelt out HR; | Thank you for these suggestions. We have made the changes accordingly, shown in track changes. We have further spelled out HR wherever it was used (additionally in Table 3). |
| Additional editor comments | |
| This is a well-written implementation research report to document the challenges and accomplishments in mitigating the COVID-19 impacts in the provision of a few essential child healthcare services in Bangladesh. It is one of few examples combining both qualitative and quantitative methods.  One issue: There are only 14 months prior to COVID-19 to establish the secular and seasonal trends. This deserves a comment on the limitations. | Thank you for your comments. We have added the following text (in bold) about the limitations of using only 14 months prior to COVID-19 to establish secular and seasonal trends (page 25 lines 488-89): “The ability to detect transient changes in EBI service delivery and coverage is limited by the shorter duration of the period of COVID-19 under investigation, **as well as the 14-month duration of time prior to COVID-19 used to establish the secular and seasonal trends**.” |

Table 1: Interrupted time series analysis of Poisson regression model of the impact of COVID-19 on primary health care services in Bangladesh

| **Primary health care services** | **Crude Incidence Rate Ratio (IRR)** | **95% CI** | **Adjusted Incidence Rate Ratio (IRR)^¥^** | **95% CI** | **P-value** |
| --- | --- | --- | --- | --- | --- |
| **Childhood vaccination** | | | | |  |
| Pentavalent vaccine doses | 0.876 | 0.873, 0.879 | 1.021 | 0.773, 1.350 | 0.880 |
| Measles-Rubella doses (MR) | 0.890 | 0.887, 0.893 | 0.981 | 0.740, 1.300 | 0.891 |
| **Maternal health services** | | | | |  |
| 4 or more antenatal care (ANC) care visits | 0.514 | 0.509, 0.520 | 0.626 | 0.516, 0.759 | p<0.001 |
| Facility-based delivery | 0.588 | 0.582, 0.594 | 0.797 | 0.673, 0.943 | 0.008 |
| Postnatal care | 0.587 | 0.582, 0.592 | 0.806 | 0.664, 0.977 | 0.028 |
| **Outpatient attendance for children** | 0.443 | 0.442, 0.444 | 0.469 | 0.312, 0.706 | p<0.001 |

Adjusted Incidence Rate Ratio^¥^: adjusted for seasonality
